# Small Cell Lung Carcinoma with Pancoast Syndrome: A Case Report

**DOI:** 10.31729/jnma.6620

**Published:** 2022-02-28

**Authors:** Sion Hangma Limbu, Narendra Bhatta, Deebya Raj Mishra, Achyut Bhakta Acharya, Avatar Verma, Rejina Shahi, Srijan Katuwal, Sunil Kumar Singh

**Affiliations:** 1 Department of Pulmonary, Critical Care and Sleep Medicine, B.P. Koirala Institute of Health Sciences, Dharan, Nepal

**Keywords:** *case report*, *pancoast syndrome*, *small cell lung cancer*

## Abstract

Small cell lung cancer mostly arises centrally in the large bronchi. The literature search revealed very limited cases of small cell lung cancer arising at the upper part of the pulmonary sulcus near the thoracic inlet as superior sulcus tumor and also manifesting with typical Pancoast syndrome. We report a case of a 71 years old male patient, presenting with features of Pancoast syndrome including Horner's syndrome with completed three cycles of chemotherapy resulting in partial response which concludes that small cell lung carcinoma has to be considered despite the clinical findings like pancoast syndrome.

## INTRODUCTION

Primary tumors in superior sulcus of the lung are broadly considered as Pancoast tumors, which account for 3% to 5% of all lung cancers.^1^ Non-small cell lung cancer (NSCLC) account for more than 95% of all Pancoast tumors whereas small cell lung cancer is considerably a rare cause.^1,2^ As per records, small cell carcinoma with Pancoast syndrome has never been reported in Nepal. We present a case of small cell carcinoma of the left lung arising in the upper lobe and eventually presenting with Pancoast syndrome: Horner's syndrome, shoulder pain and, wasting, paraesthesia and weakness of the upper limb.

## CASE REPORT

**Figure 1:**
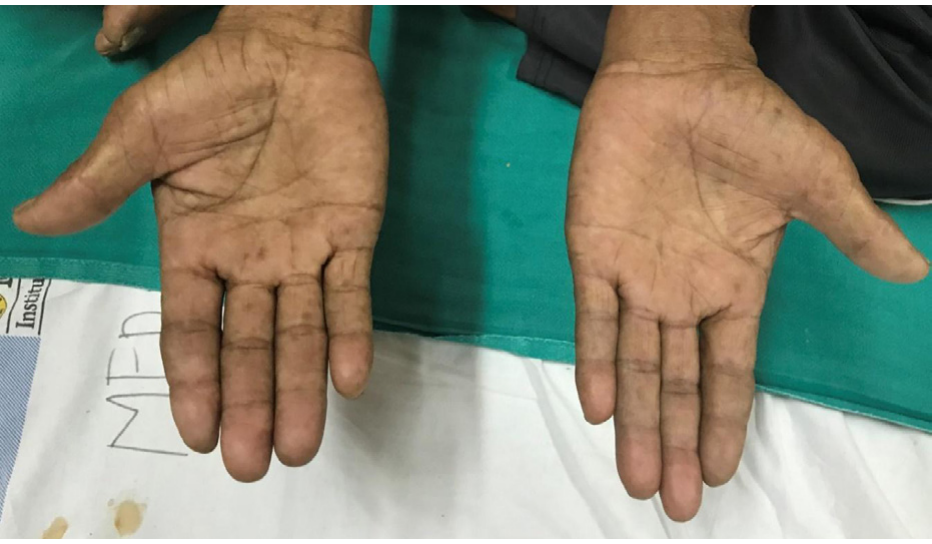
Wasting of left hypothenar hand muscles.

**Figure 2:**
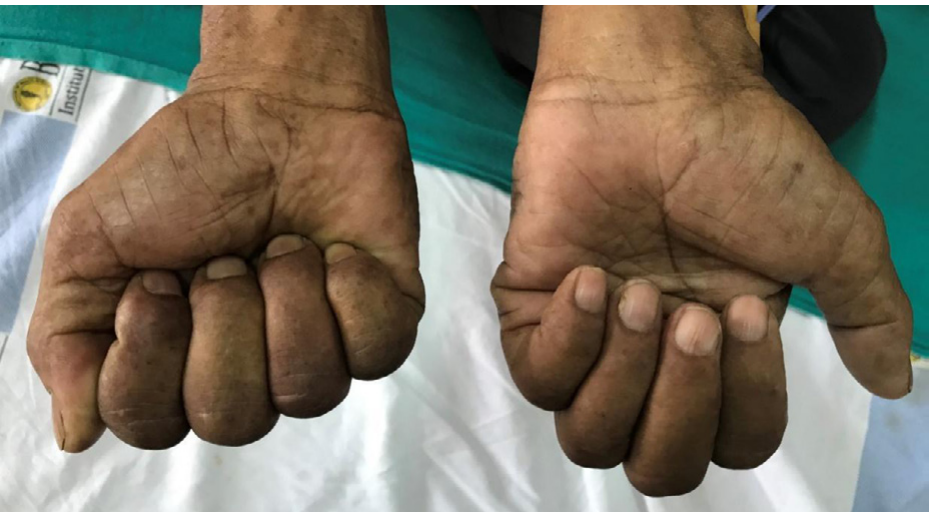
Partial closure of left hand while intending to make a fist (involvement of C8, T1).

A case of a 71 years old Asian male, a farmer, chronic smoker for 55 years (one pack of cigarettes including marijuana per day: approximately 55 pack years), presented with intermittent cough for 3 months, hoarseness of voice for 2 months and painful swelling over left cervical and supraclavicular region for 2 months. He also complained of constant, aching pain in his left shoulder which progressed to involve the axilla and upper arm, and was associated with tingling sensation over the left hand. Physical examination revealed bilateral clubbing and left supraclavicular lymph nodes that were hard and matted. Bilateral vesicular breath sounds were heard. Neurological examination showed wasting and weakness of left-hand muscles (Figure 1), and inability to completely close the left hand (Figure 2).

**Figure 3a:**
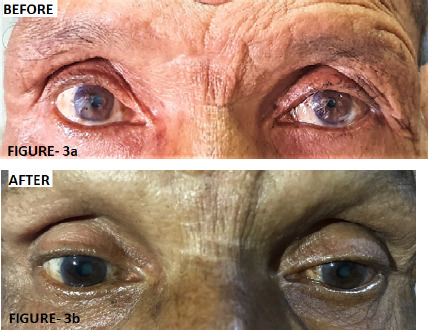
Horner's Syndrome (left sided drooping of eyelid and miosis). **Figure 3b.** Improvement in miosis and drooping of left eyelid after three cycles of chemotherapy.

After admission for diagnostic workup and on detailed examination, the patient manifested Horner's syndrome (Figure 3).

**Figure 4:**
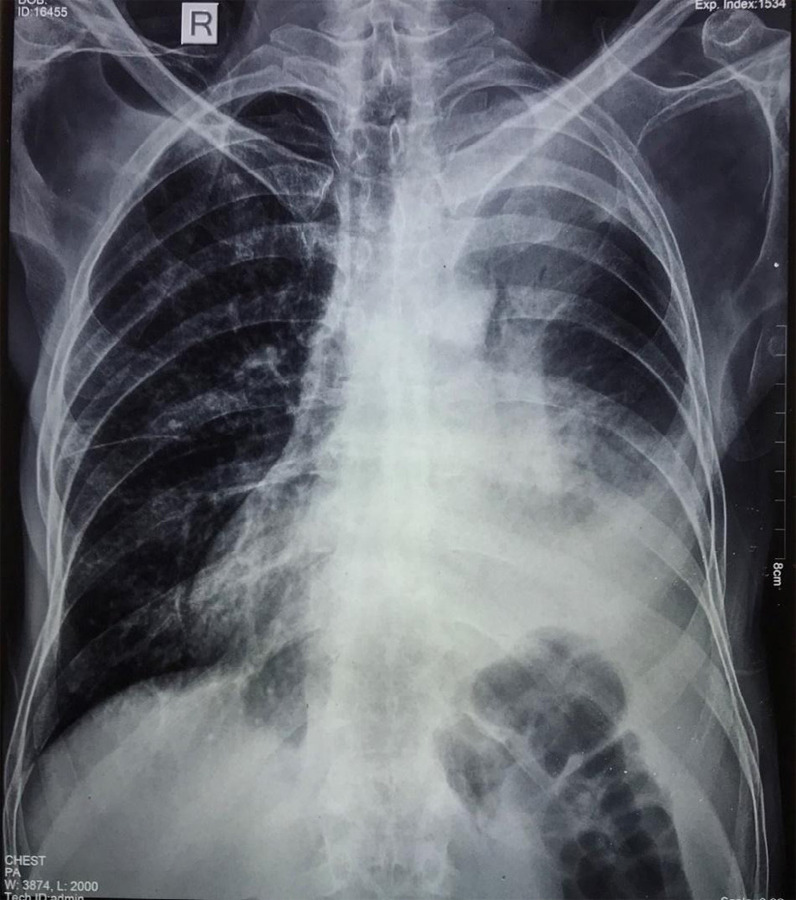
Left lung mass with regular borders in the apical region and moderate pleural effusion.

Sputum Acid Fast Bacilli (AFB) was negative and the biochemical and hematological parameters were within normal limits. Chest imaging showed a mass with regular borders in the left superior sulcus with moderate left sided pleural effusion (Figure 4).

**Figure 5:**
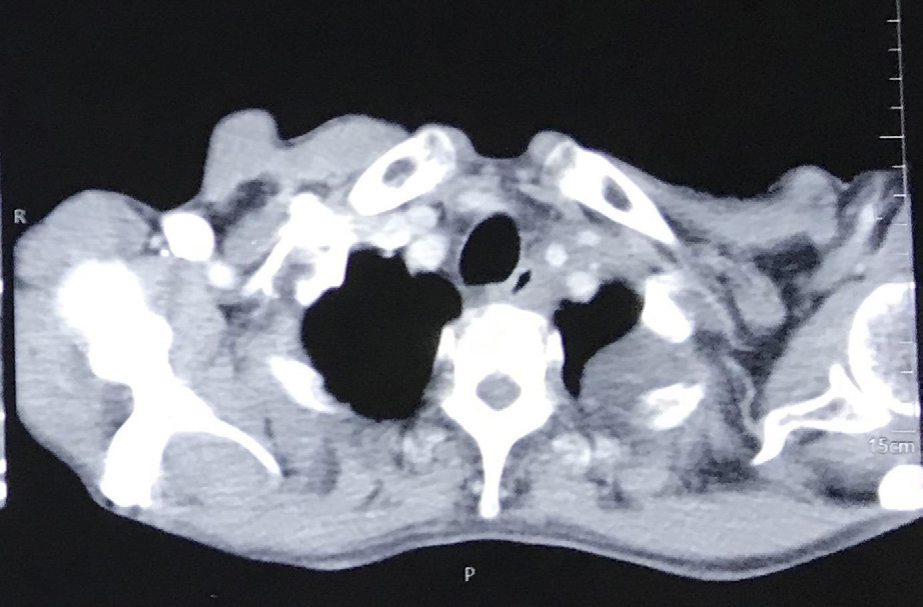
Pancoast superior sulcus tumor.

Contrast Enhanced Computed Tomography (CECT) chest report showed heterogeneously enhancing soft tissue density lesion of approximately 5.4x5.2x4.4cm with lobulated irregular outline with spiculations involving left upper lobe, continuous anteriorly with a mass of enlarged mediastinal lymph nodes at pre-vascular area extending superiorly up to the supraclavicular area (Figure 5).

As per plan, the patient underwent Ultrasonography (USG) guided lymph node biopsy. Imprint slide was prepared and sent for investigation. The cervical and supraclavicular lymph node biopsy report was suggestive of highly cellular small cell lesions with marked karyorrhexis. The cytopathology report showed overall features of metastatic deposits of small cell carcinoma. Repeated cytology of pleural effusion did not reveal malignant cells. A diagnosis of bronchogenic carcinoma left lung, small cell carcinoma: clinical stage of T4N3M0, limited stage III A was suggestive. He received chemotherapy: carboplatin (because of old age and poor performance status at the time of presentation) and etoposide of three cycles within a period of 2 months. After three cycles of chemotherapy, there was 54% reduction in tumor volume. On Response Evaluation Criteria in Solid Tumors (RECIST) 1.1 criteria scale, it was suggestive of partial response. Clinically, his left upper limb symptoms and Horner's syndrome have significantly improved. After the fourth cycle of chemotherapy the patient is planned for sequential radiotherapy.

## DISCUSSION

A few handful cases of Pancoast syndrome secondary to small cell lung cancer have been described in the literature and only one could be found presenting with Horner's syndrome as well.^3^ Most Pancoast tumors constitute Non-small cell lung cancers, adenocarcinoma being the most common histological type according to the recent studies (squamous cell carcinoma used to be the most common).^4^ Lung adenocarcinoma starts in glandular cells and tends to develop in smaller airways, like alveoli. Therefore, adenocarcinoma is usually located along the periphery of the lungs and is more likely to present as Pancoast syndrome. On the other hand, SCLC usually begins centrally in the major bronchi. Thus, SCLC presenting as Pancoast syndrome is a rare condition.

This patient had presented with typical Pancoast syndrome including Horner's syndrome. Although the location of the tumor was suggestive of non-small cell origin, lymph node biopsy cytopathology was fairly able to diagnose the case as SCLC. SCLC has the poorest prognosis among all histological types of lung cancer, with a tentative 5 years survival rate ranging from approximately 25% for limited disease to 1-5% for extensive disease.^5^ SCLC is aggressive with a short doubling time and high mitotic rate. Approximately 60% of patients present with metastatic disease (most common metastatic sites include the brain, liver, bone and adrenal glands). If left untreated, SCLC is characterized by rapid tumor progression with a median survival of mere 2 to 4 months. Patients with limited-stage are suitable candidates for curative-intent radiation therapy and chemotherapy whereas those with extensive-stage are often treated with chemotherapy; radiation is reserved for selective candidates.^6^

After receiving three cycles of carboplatin 550mg and etoposide 170mg, this patient showed a partial tumor regression according to RECIST criteria. His left upper arm pain has completely subsided and now can move his arm above his shoulder, making an almost complete fist although the tingling sensation still remains. After 3 months of presentation of his symptoms and having received his fourth cycle of same regimen chemotherapy lately, the patient has clinically improved and therefore, is fairly satisfied with the treatment. This case points towards keeping in mind the possibility of SCLC even in the cases presenting as Pancoast syndrome.
